# Microalgae Produce Antioxidant Molecules with Potential Preventive Effects on Mitochondrial Functions and Skeletal Muscular Oxidative Stress

**DOI:** 10.3390/antiox12051050

**Published:** 2023-05-05

**Authors:** Jordi Vignaud, Céline Loiseau, Josiane Hérault, Claire Mayer, Martine Côme, Isabelle Martin, Lionel Ulmann

**Affiliations:** BiOSSE (Biology of Organisms, Stress, Health, Environment), Institut Universitaire de Technologie, Département Génie Biologique, Le Mans Université, F-53020 Laval, France; jordi.vignaud@univ-lemans.fr (J.V.); celine.loiseau@univ-lemans.fr (C.L.); josiane.herault@univ-lemans.fr (J.H.); claire.mayer@univ-lemans.fr (C.M.); martine.come@univ-lemans.fr (M.C.); isabelle.martin@univ-lemans.fr (I.M.)

**Keywords:** microalgae, antioxidant molecules, redox homeostasis, oxidative stress, mitochondrial function, musculoskeletal diseases, exercise

## Abstract

In recent years, microalgae have become a source of molecules for a healthy life. Their composition of carbohydrates, peptides, lipids, vitamins and carotenoids makes them a promising new source of antioxidant molecules. Skeletal muscle is a tissue that requires constant remodeling via protein turnover, and its regular functioning consumes energy in the form of adenosine triphosphate (ATP), which is produced by mitochondria. Under conditions of traumatic exercise or muscular diseases, a high production of reactive oxygen species (ROS) at the origin of oxidative stress (OS) will lead to inflammation and muscle atrophy, with life-long consequences. In this review, we describe the potential antioxidant effects of microalgae and their biomolecules on mitochondrial functions and skeletal muscular oxidative stress during exercises or in musculoskeletal diseases, as in sarcopenia, chronic obstructive pulmonary disease (COPD) and Duchenne muscular dystrophy (DMD), through the increase in and regulation of antioxidant pathways and protein synthesis.

## 1. Introduction

The development of new food supplements has aimed to prevent age-related disorders, cardiovascular disease and improve muscle performance or well-being [1,2,3]. Seafood, and particularly oily fish, is an important source of long-chain polyunsaturated fatty acids (LC-PUFAs), with beneficial effects on human health [4]. LC-PUFAs present in fish are provided by microalgae, a vast group that belongs to the phytoplankton. Microalgae are the most important part of aquatic ecosystems [5]. These photosynthetic microorganisms are a promising source of many bioactive molecules, such as fatty acids, steroids, carotenoids, polysaccharides, lectins, mycosporine-like amino acids, halogenated compounds, polyketides, toxins, agar–agar, alginic acid and carrageenan [6]. Microalgae contain various compounds with demonstrated potential for human health and medicine. The therapeutic properties of microalgae exhibit a large range of applications, such as in cardiovascular health, anticancer, anti-inflammatory, anticoagulant, antiviral, antibacterial or antifungal human medicinal products. Many bioactive compounds from microalgae have strong beneficial properties that effectively reduce the production of inflammatory compounds against muscle breakdown [7].

Muscle is a dynamic tissue that is rich in mitochondria, the primary function of which is to maintain the supply of ATP through oxidative phosphorylation, I order to facilitate movement. It is an important consumer and producer of essential metabolites, when challenged by aerobic or resistance exercise. This is essential for a healthy life and to prevent diseases such as Alzheimer’s disease, cancer or ageing [8,9]. However, during muscle diseases or traumatic exercises, mitochondria produce metabolites generating oxidative stress (OS) [10]. OS is defined as a chronic imbalance in the pro-oxidant species derived from oxygen and nitrogen (RONS) and antioxidant systems, resulting in increased oxidative damage to various cell components, as well as an alteration of numerous signaling pathways.

The aim of this review is to identify potential source of antioxidant biomolecules from microalgae (biomass and extracted molecules). In parallel, mitochondrial function and oxidative stress associated with exercise or muscle diseases are developed. Then, the preventive effects of microalgal biomass and its bioactive molecules against mitochondrial dysfunction and oxidative stress in muscle are presented.

## 2. Microalgae as Sources of Molecules with Antioxidant Effects

Although the classification of algae and microalgae may change according to the evolution of analytical tools, these photosynthetic organisms can be broadly classified into red (*Rhodophyta*), brown (*Phaeophyta*) and green algae (*Chlorophyta*) based on their characteristics [11]. Indeed, microalgae are divided into different categories according to their pigment content, morphological distinctions (spherical, elliptic, rod-like, and fusiform cells) and the existence of thorns, cilia and flagella, among other characteristics. Further, they are also grouped based on their size: picoplankton (0.2–2 µm), nanoplankton (2–20 µm) and microplankton (20–200 µm) [12].

**Figure 1:**
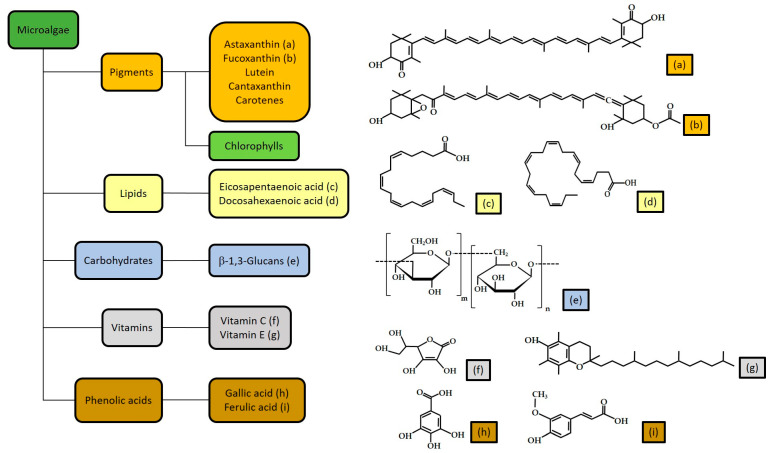
Microalgal bioactive molecules [15,16].

Microalgae are unicellular organisms found in aquatic environments. They play a key role in marine ecosystems, forming the basis of the food web as primary producers. These photosynthetic micro-organisms are regarded as one of the best renewable resources for numerous medicinal compounds due to their richness in primary and secondary bioactive metabolites, such as carbohydrates, proteins, lipids, vitamins, pigments, LC-PUFA, polyphenols and other chemicals (Figure 1). Among the various microalgal genera, *Spirulina*, *Chlorella*, *Haematococcus*, *Dunaliella*, *Nannochloris*, *Botryococcus*, *Phaeodactylum*, *Porphyridium*, *Chaetoceros* and *Skeletonema* are the more widely used microalgae, with commercial value due to their diverse content of therapeutic bioactive compounds [13].

Among molecules produced by microalgae, many are known to have antioxidant activities. These molecules include vitamins, such as vitamins A, C and E, polyphenols, carotenoids and bioflavonoids. Antioxidants are well known to provide health benefits and play an essential role in cell protection from the effects of free radicals. In the context of an increasing demand for these high-value-added products, microalgae are undoubtedly an interesting solution. The antioxidant power of microalgae is equal to or higher than the antioxidant activity of higher plants or fruits. Indeed, in *Chlorophyta* and *Eustigmatophyceae*, the antioxidant potential can reach 260 Trolox equivalents μmol·g^−1^ dry matter (DM), while in raspberry, the antioxidant activity reaches 224 Trolox equivalents μmol·g^−1^ DM [14]. Thus, for some years now, marine microalgae have been considered a potential source of high-value-added biomolecules, such as antioxidant compounds.

### 2.1. Pigments

Microalgae produce a wide variety of pigments, such as chlorophylls, carotenoids and flavonoids. Among the microalgae, *Botryococcus braunii*, *Chlorella* sps. *Chlorococcum* sp., *Coelastrella striolata*, *Haematococcus pluvialis*, *Dunaliella salina*, *Nanochloropsis* sps., *Scenedesmus* sps., *Spirulina platensis* and some diatoms are known to produce β-carotene (β-Car), lutein, canthaxanthin, astaxanthin (Asx) and fucoxanthin (Fcx) [17].

Microalgae are the most promising sources of natural carotenoids. Carotenoids are fat-soluble molecules that constitute a class of terpenoid pigments, derived from a 40-carbon polyene chain, which can be considered their molecular backbone. Carotenoids are divided into carotenes and xanthophylls. They are mainly present in the pigment–protein complexes within the membrane of thylakoids, but some microalgal species can also accumulate carotenoids (β-Car and Asx) in lipid globules located in the stroma of the chloroplast or in the cytoplasm [18]. Among different microalgae, the total carotenoid content ranges from 3.04 mg·g^−1^ dry weight (DW) for *Scenedesmus almeriemis*, to 35 mg·g^−1^ DW for the hypersaline species *Dunaliella salina* [19]. The carotenoids of *Dunaliella salina* are mainly represented by β-Car (98.58% of total carotenoids) [19]. Under optimized culture conditions, the green microalga, *Asterarcys quadricellulare,* was shown to produce 47.0, 28.7, 15.5 and 14.0 μg of β-Car, lutein, Asx and canthaxanthin mg^−1^ of dry biomass, respectively [20].

In diatoms, Fcx is one of the main pigments present in chloroplasts. Its production varies from 0.82 mg·g^−1^ DW for *Phaedactylum tricornutum* to 26.6 mg·g^−1^ DW for the microalga *Mallomonas* sp. [21]. The Fcx content depends on microalgal species, the culture conditions and the extraction process. In the review by Khaw, the Fcx content is described to vary between species and within species. Indeed, in the microalga *Isochrysis galbana*, the Fcx content varies between 0.22 and 1.82% of the DW [22]. Conversely, some microalgal species produce very low amounts of Fcx, such as *Phaeodactylum tricornutum* (0.01% DW) and *Skeletonema costatum* (0.04% DW). In the different species studied, the highest Fcx content after optimization of the culture conditions was found in *Tisochrysis lutea,* with a percentage of 7.94% DW [22].

In the literature, Asx is described as the pigment with the highest antioxidant activity. *Haematococcus lacustris* (*Chlorophyceae*) is the main source of Asx, accumulating up to 7% of its DW [23]. Although *H. lacustris* is able to accumulate a significant amount of Asx, it has been shown that in the green alga, *Chlorella zofingiensis*, the Asx content can reach 6.8 mg·g^−1^ DW under stress conditions (high light, nitrogen deprivation and salinity stress) [24].

### 2.2. Vitamins

Microalgae are also a source of vitamins, such as vitamins C and E.

In microalgae, ascorbic acid, or vitamin C, is mainly present in the cytosol and chloroplasts. The vitamin C content varies from 0.06 mg·g^−1^ DW for the genus *Skeletonema*, to 18.79 mg·g^−1^ DW for the genus *Chaetoceros*. For the same genus, there is a great variability in the vitamin content. For example, for the microalga *Chaetoceros*, the ascorbic acid content varies from 0.12 to 18.79 mg·g^−1^ DW [25].

In the literature, few publications report the tocopherol (TOC) composition of microalgae. TOC or vitamin E is a liposoluble antioxidant only synthesized by photosynthetic organisms and is located in the membrane lipid bilayers, mainly in those of chloroplasts. Vitamin E is present in microalgae such as *Dunaliella tertiolecta*, *Tetraselmis suecica*, *Nannochloropsis oculata*, *Chaetoceros calcitrans* and *Porphyridium cruentum*. The total TOC content varies greatly between species, with the highest amount found in *Tetraselmis* sp. (6320 µg·g^−1^ DW) and the lowest in *Phaeodactylum tricornutum* (13.12 µg·g^−1^ DW) [26].

### 2.3. Polysaccharides

Polysaccharides are polymers made up of osidic units linked by glycosidic bonds. Microalgae are shown to contain several polysaccharides with antioxidant activity. However, this activity remains rather weak.

Beta-1,3-glucans, also named laminarin, chrysolaminarin or mycolaminarin, depending on the algal species, are one of the most abundant polysaccharides in microalgae [27]. They are involved in carbon storage and constitute the structural components of the cell walls of these organisms. The flagellate alga, *Poterioochromonas malhamensis*, is a potential source of β-1,3 glucan. This polysaccharide content makes up to 55% of its biomass [28]. In 2016, Schultze et al. studied the composition of metabolites, and in particular β-glucans, in 47 microalgae [29]. Under standard culture conditions, the β-glucan content varies from 1.7% to 24.8% of DW. *Phaeodactylum tricornutum* and *Mesotaenium caldariorum* have a very low content (1.7% DW), while *Scenedesmus ovalternus* has the highest accumulation of β-glucans, with 24.8% of the DW.

In diatoms, chrysolaminarin, composed of β-1,3-glucan with β-1,6-branched chains, is the main form of polysaccharide storage. This polysaccharide is present at high levels in diatoms such as *Odontella aurita* (up to 63% DW), *Phaeodactylum tricornutum* and *Thalassiosira pseudonana* (up to 23% DW) [30].

### 2.4. Phenolic Compounds

Phenolic compounds constitute a large family of antioxidant molecules present in higher plants, macroalgae, and more recently studied in microalgae. Approximately 8000 structures of phenolic compounds have been identified. All contain one or more aromatic rings with one or more hydroxyl substituents. Various studies have been carried out on the total phenol content of different microalgal strains and in different geographical locations. A recent study by Almendinger et al. (2021) showed that two microalgal species, *Neochloris oleobundans* and *Wilmottia murravi*, contain high levels of phenolic compounds (>20 mg gallic acid eq·g^−1^), out of the 13 microalgae studied [31]. In a study of Leon-Vaz et al. (2023), 19 species of nordic microalgae were cultivated in standard conditions and under high light and cold stress conditions to explore their ability to produce bioactive compounds such as polyphenols [32]. The strains *Chlorococcum* sp. and *Scenedesmus* sp. produced higher concentrations of phenolic compounds during stress. The green alga, *Chlamydomonas reinhardtii*, was able to increase the production of polyphenols during a high light exposure [33]. Anwer et al. (2022) determined the total phenol content from several microalgal species using different types of extraction. They found that *Spirulina* sp. contained more total phenolic compounds than the other species [34]. Trentin et al. (2022) showed that two microalgae, a naviculoid diatom and a green microalga (*Tetraselmis marina*), are rich in polyphenolic compounds. Thus, all these microalgae can be considered good candidates to serve as sources of these high-value compounds [35].

### 2.5. Omega-3 Polyunsaturated Fatty Acids

Microalgal lipids are characterized by a large diversity of fatty acid profiles. Several studies on the microalgal fatty acid composition of lipids have revealed the presence of large amounts of LC-PUFAs. LC-PUFAs belong to the omega-3 series (ω3-PUFA). This is the case for docosahexaenoic acid (C22:6 ω3, DHA) and eicosapentaenoic acid (C20:5 ω3, EPA), of which the beneficial effects on health are now widely recognized.

Among the microalgae producing ω3-PUFAs, a distinction can be made between those synthesizing only EPA or DHA, and those synthesizing EPA and DHA, in proportions that vary according to the species and the culture conditions (culture regimen, temperature, salinity, light, pH and nutrients). The lipids of bacillariophytes (or diatoms) are characterized by high levels of EPA and low or even zero levels of DHA [36]. A recent study carried out a screening of new strains of microalgae producing bioactive substances, and highlighted the particular interest of diatoms as EPA-producing organisms. Of the nine selected species, *Thalassiosira weissflogi* had the highest proportion of EPA (21.4% of total fatty acids) and an absence of DHA [37]. DHA is specific to dinoflagellate and haptophyte lipids. Dinophytes can produce large amounts of DHA, with up to 40% of total fatty acids in some taxa. In haptophytes, DHA production can reach 30% of total fatty acids [36]. Specifically, *Crypthecodinium cohnii*, a heterotrophic dinoflagellate, is able to produce DHA at high levels (up to 50% of DW), while EPA is totally absent [38].

## 3. Muscular Mitochondrial Function and Oxidative Stress during Exercise

### 3.1. Muscular Protein Balance

Muscle quality and mass are mainly regulated by a balance between protein synthesis and degradation. The protein pool is maintained by a fine balance between muscle protein synthesis (MPS) and muscle protein breakdown (MPB). These processes respond to nutrients and contractile activity actions affecting physical performance, injury prevalence and disease [39,40,41].

#### 3.1.1. Muscle Protein Synthesis

One of the characterized regulators of muscle protein translation is the mammalian target of rapamycin (mTOR) [42]. There are two complexes of mTOR: mTORC1 and mTORC2, but only the former is involved in MPS [43]. MPS is regulated by IGF1 (insulin-like growth factor 1)/PI3K (phosphoinositide 3-kinase)/Akt (protein kinase B)/mTOR pathways, with mTORC1 being able to control protein translation [44,45]. The target of mTORC1 results in the regulation of the level of phosphorylation of eukaryotic translation initiation factor 4E-binding protein 1 (4E-BP1) protein. This factor is a repressor of eukaryotic translation initiation factor 4E (EIF4E). The target of mTORC1 also results in the phosphorylation of ribosomal protein S6 kinase beta-1 (p70S6K), which in turn leads to the activation of eukaryotic translation initiation factor 4B (EIF4B). Moreover, p70S6K activates the ribosome biogenesis and an over-time translation through eukaryotic elongation factor-2 kinase (EEF2K), which also contributes to the activation of MPS [46,47]. In healthy people, MPS is constant, negatively regulated by AMP-activated protein kinase (AMPK) during exercise and positively after exercise [48]. In contrast, during muscle disease associated with mitochondrial dysfunction, AMPK suppresses the MPS via an inhibition of mTORC1 [49].

#### 3.1.2. Muscle Protein Breakdown

In opposition to MPS, MPB is activated during exercise. There are two major types of MPB: autophagy and the ubiquitin proteasome system (UPS).

Autophagy is a breakdown system of proteins, energy substrates, mitochondria and other organelles [50,51]. It plays an important role in muscle homeostasis when mitochondria or misfolded proteins are not eliminated. This process leads to a disorganization of the sarcomere and induces muscle atrophy [52]. Autophagy follows four steps: induction, nucleation, elongation and substrate isolation, and fusion with lysosomes [53]. Its activation is associated with a variety of stress factors such as OS, hypoxia or protein aggregation. The pre-autophagosome formation is controlled by the protein complexes ULK-ATG13 and 101-Fip200 [53,54]. Moreover, mTORC1 is a key regulator of autophagy through the direct phosphorylation of ULK [55]. Then, the fusion of lysosomes with autophagosome degrades its content and releases amino acids [56].

The UPS targets the proteins contained in the mitochondria, cytosol, nucleus and endoplasmic reticulum [57]. The UPS is an ATP-dependent non-lysosomal protein degradation mechanism in cells [53]. There are three types of ubiquitin enzymes: E1 (ubiquitin activating enzyme), E2 (ubiquitin conjugating enzyme) and E3 (ubiquitin ligase), that transfers the protein to the autophagosome [58].

### 3.2. Mitochondrial Function and RONS Production

Mitochondria are cell organelles known to be involved in muscle physiology, their activity being modified during muscle damages due to pathologies or physiological modifications.

**Figure 2:**
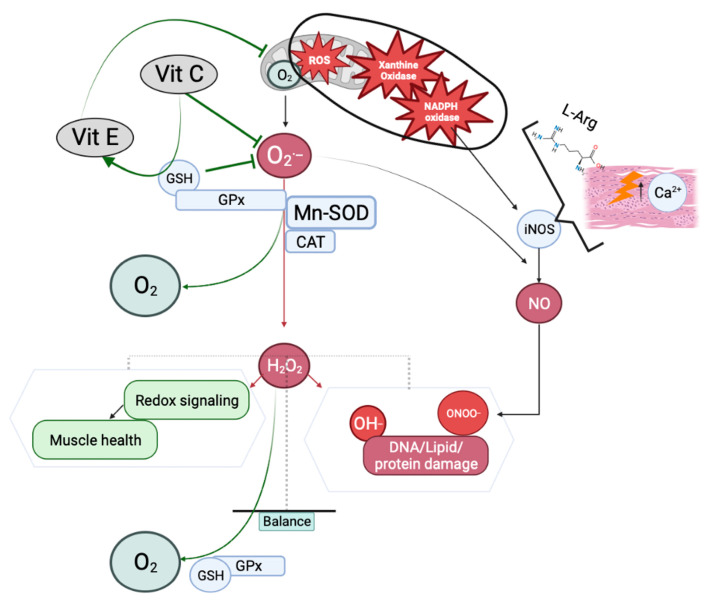
RONS production and cellular fate. The mitochondrial function produces superoxide anion O_2_^·−^, which is transformed into H_2_O_2_ due to the antioxidant enzymes Mn-SOD, CAT and GPx. Muscular damage, oxidation of L-arginine (L-Arg) and increased calcium level (↑) activates iNOS and the production of NO. An overproduction of H_2_O_2_ or the action of O_2_^·−^ with NO will induce OS through OH^−^ and ONOO^−^, respectively, resulting in DNA, lipid and protein damage. Contrarily, a controlled H_2_O_2_ level will be in favor of muscle health. Non-enzymatic antioxidants such as vitamins C and E inhibit the production of O_2_^·−^, and thus also limit the oxidant stress. Created using Biorender.com (accessed on 28 April 2023).

Besides the role of muscle mitochondria in ATP synthesis and oxidation of numerous substrates, mitochondria are involved in other mechanisms, such as apoptosis induction [59,60,61], free radical production [62,63], calcium homeostasis [64] or thermogenesis regulation [65]. According to muscle physiology, the production of ATP can be provided through two mechanisms: the anaerobic glycolysis and the oxidative phosphorylation. The oxidation of substrates by the oxidative phosphorylation pathway is a key factor for muscles during endurance training [66,67,68], with an increase in superoxide anion (O_2_^·−^) content in contracting muscle fibers [67,69]. The RONS are composed of the reactive oxygen species (ROS), among which O_2_^·−^ is produced by oxidase xanthine [70], nicotinamide adenine dinucleotide phosphate (NADPH) oxidase and mitochondria [71], or the hydrogen peroxide (H_2_O_2_) that is produced by reaction between O_2_^·−^ and the mitochondrial superoxide dismutase 2 (Mn-SOD) [72,73]. H_2_O_2_ is able to cross membranes and act on cell proteins. In response to the increased RONS level in cells, an enzymatic cascade involving peroxidase glutathione (GPx), catalase (CAT) and peroxidase thioredoxin (TPx) will convert H_2_O_2_ into H_2_O or a hydroxyl group (OH^−^) that is able to oxidize DNA, lipids and proteins [72,73] (Figure 2). Among the RONS, there are also reactive nitric species (RNS). In physiological conditions, they have an important role in skeletal muscle signaling [74]. Nitric oxide (NO) is mainly produced via the oxidation of the amino acid L-arginine, in a highly controlled process requiring oxygen, NADPH and other cofactors. This reaction is catalyzed by specialized nitric oxide synthases (NOSs), of which the activities depend on the intracellular calcium level [75]. The role of NO is to maintain glucose uptake, vascular perfusion during contraction, mitochondrial function and the excitation–contraction coupling. Thus, muscle diseases associated with mitochondrial dysfunction generate proinflammatory conditions resulting in inducible NOS (iNOS) activation and an overproduction of NO. Then, NO and O_2_^·−^ produce peroxynitrite (ONOO^−^) and nitrogen species [75].

### 3.3. Inflammation Pathways Due to the RONS

Exhaustive exercise produces proinflammatory cytokines involved in the transcriptional regulation of redox signaling, through the activation of nuclear factor (erythroid-derived 2)-like 2 (Nrf2) and nuclear factor-kappa B (NF-κB) signaling pathways [76]. Exercises increase the release of muscle interleukin (IL)-6, which can negatively regulate the tumor necrosis factor alpha (TNF-α) and stimulate IL-10 secretion, two mechanisms resulting in macrophage M2 activity improvement, that increases the anti-inflammatory response [77]. During low systemic conditions, it has been shown that exercise exerts an anti-inflammatory effect and prevents chronic disease associated with OS, mitochondria dysfunction and protein imbalance [78,79].

In inflammation-associated muscle diseases, it is reported that NF-κB/TNF-α/IL-6 pathways induce an increase in the proinflammatory macrophage M1, which results in muscle atrophy [80,81]. The RONS production is autoregulated by NF-κB and TNF-α [81,82]. The factor Nrf2 is a negative regulator of NF-κB, which can reduce inflammation during exercise, and for which a stimulation occurs following RONS production [83].

### 3.4. Antioxidant Responses to RONS Production

During physical activities, the overproduction of ROS induces damage to skeletal muscle, but this production can be regulated by the antioxidant defense system. The use of antioxidant supplements, such as vitamins E and C, can also be used to prevent ROS damage. The use of these vitamins and other compounds, such as resveratrol and coenzyme Q10, has been reported to have several effects on skeletal muscle during adaptations to training, inflammation or muscle damage [84].

To optimize RONS production, two possibilities have been reported in the scientific literature: the identification of an antioxidant mix with defined concentrations [85,86] and the use of a new form of antioxidant, also named secondary antioxidant, which interact with antioxidant response elements (AREs) of genes rather than activating RONS scavengers. Among them, resveratrol showed a positive role in AREs during physical activity [87,88], and ergothioneine, a sulfated derivate of histidine, was shown to stimulate the gene Nrf2, an important regulator of the ARE [89]. Ergothioneine is the only sulfur-derivate of histidine to be reported to have any effect on muscle, but it can be noticed that ovothiols, other derivates of histidine produced by microalgae [90,91] have also been reported to regulate Nrf2 into keratinocytes, inducing a dermo-protective effect [92].

The antioxidant activity can be carried out by non-enzymatic molecules, such as vitamins C and E, or by enzymes [93]. When considering enzymes, the SOD family is able to neutralize O_2_^·−^. The cytosolic SOD activity is around 65–85% of the total cell SOD activity, while in mitochondria, it is around 15–35% [70]. Moreover, the SOD activity is higher in type 1 fiber (slow twitch) than in type 2 (fast twitch) [94]. This mechanism cannot be completed without a synergy with CAT and GPx that converts reduced glutathione (GSH) into oxidized glutathione (GSSG). The levels of GSH are higher in type 1 fiber than in type 2 [95]. The GSH–GSSG ratio is a marker of OS, in relation to the increase in H_2_O_2_ [96,97] (Figure 2).

Vitamin C is an important antioxidant compound because it rapidly gives its electron and prevents the overproduction of RONS [98]. Vitamin E or TOC, including α-TOC, is the second most consumed vitamin. Its metabolism is regulated by the liver and this vitamin has a strong antioxidant activity [99]. However, studies report that long-term vitamin E supplementation may increase the risk of heart failure in patients with vascular disease or diabetes mellitus [100].

### 3.5. Muscular Redox Balance and Mitochondrial Adaptation

High intensity exercise causes damage in muscles, which is repaired by protein turnover. However, exercise also generates OS and inflammation that have to be managed [10]. Numerous athletes use antioxidant supplementation to counteract the overproduction of RONS and inflammation during exercise [101]. However, antioxidant supplementation must be carefully used due to the role of RONS in muscle adaptation [85]. During aerobic exercise, RONS is able to stimulate peroxisome proliferator-activated receptor-γ coactivator (PGC1-α) with, as a consequence, an improvement in mitochondrial biogenesis and muscle adaptation [102]. In the Merry and Ristow review, it is explained that high levels of vitamins C and E prevented the activation of PGC1-α, limiting muscle adaptation and maximal oxygen consumption during aerobic exercise [103]. The same observations have been made during resistance exercise. Indeed, after 10-week strength training protocol in women, the gains in peak torque and total work were lower in the group with vitamin C and E supplementation than in the control group [104].

## 4. Muscular Diseases and Overproduction of RONS

Without being exhaustive, the most important pathologies reported in the literature and correlated with an important RONS production are sarcopenia, chronic obstructive pulmonary disease (COPD) and Duchenne muscular dystrophy (DMD). The OS conducts muscle atrophy with drastic consequences to human life. The aim of this section is to describe the mechanisms that result from OS and the need for antioxidants to prevent or improve the wellness of affected persons.

### 4.1. Sarcopenia

During ageing, a general muscle deconditioning, named sarcopenia, occurs. This syndrome is characterized by a progressive and general loss of skeletal muscle mass and strength, with a risk of adverse outcomes such as physical disability, poor quality of life and death [105]. Inactivity is the most common cause. In fact, from 50 years of age onward, muscle mass decreases by 1–2%/year and strength by 1.5–5%/year [106]. Moreover, ageing decreases the amount of type 2 muscle fibers, resulting in a dominance of type 1 muscle fiber and a higher mitochondrial activity [107].

Two stages of sarcopenia are identified: due to age or ageing and when associated with disease or disability. In both stages, the cross-sectional area of each fiber decreases as a consequence of a imbalance in protein turnover [108] and an exacerbation of myonuclear apoptosis [109]. These two phenomena can result from increased ROS production [110] and decreased antioxidant systems in skeletal muscle mitochondria [111]. Indeed, RONS can damage the protein structure [112]. Moreover, clinical and animal studies have shown an increase in protein carbonylation and lipid peroxidation [111,113]. In response to RONS production during sarcopenia, mitochondrial Mn-SOD and GPx activities increase to regulate H_2_O_2_ and O_2_^·−^ productions [114,115,116]. In the study of Shenton et al. (2006), the low levels of H_2_O_2_ appear to stimulate protein synthesis, but when in excess, they altered the protein synthesis by mRNA blocking at the ribosome level [117]. In aortic smooth muscle cultured cells, it has been reported that H_2_O_2_ impaired the mTOR complex and blocked the phosphorylation of 4E-BP1 and p70S6K [118]. In aging rats, it has been reported that the decrease in IGF-1 and Akt phosphorylation can reduce mTOR [119]. Finally, an altered redox status improves inflammation and protein degradation, with an increase in TNF-α, muscle ring finger-1 (MuRF-1), muscle atrophy F-box (atrogin-1) and proteasome activity [119,120].

### 4.2. Chronic Obstructive Pulmonary Disease

Smoking has been shown to be the first cause of COPD. Respiratory obstruction results in a decrease in oxygen transport in peripheral muscles [121]. It appears that the lower limb muscles are more affected by this oxygen depletion [122]. Moreover, previous studies have shown a decrease in strength and endurance, and severe muscle fatigue [123,124]. All these findings are partly responsible for a muscular atrophy [125], with a decrease in type 1 fibers and an increase in type 2 fibers [126].

From a bioenergetic point of view, mitochondrial density is decreased and glycolytic metabolism seems to be more stimulated in quadriceps muscle [127,128]. Concerning MPB, an increased level of atrogin-1 has been reported, but the literature is controversial about the increase in the expression of MuRF-1 and LC3B (microtubule associated protein 1 light chain 3 beta) levels [125,129]. In the IGF1/PI3K/Akt/mTOR pathways, the increase in IGF-1 and Akt associated with an increased expression of 4E-BP1, a target of mTOR, resulted in an inhibition of MPS [130,131]. Finally, in COPD, H_2_O_2_ and O_2_^·−^ levels are increased in blood circulation, but also in skeletal muscle. Moreover, as mitochondria are considered the first source of H_2_O_2_ production in COPD patients by comparison with healthy persons, exercise has been reported to improve OS, particularly in the Complex III of respiratory chain [132,133].

Several studies have demonstrated an increase in antioxidant enzymes such as SOD, to compensate for the redox imbalance [134]. Here, as in sarcopenia, the relation between ROS and muscle atrophy is well established [135]. Indeed, an increased ROS level is known to stimulate proteolysis through the UPS, autophagy or calpain/caspase pathways [136].

### 4.3. Duchenne Muscular Dystrophy

DMD is a genetic disorder characterized by progressive muscle degeneration and weakness due to dystrophin alterations. Opposingly to other muscle pathologies, DMD is diagnosed during childhood, usually between 4 and 5 years of age [137,138]. This disease mostly affects boys (1 in 3500–6000 boys), but in rare cases, it can also affect girls. Indeed, X-chromosome-linked muscle disease is caused by dystrophin gene mutations [139]. Dystrophin binds the cell membrane to the actin filament. The lack of dystrophin leads to mechanical fragility and damage during muscle contraction [140].

To improve the therapy approach, several studies are conducted to develop natural antioxidant and anti-inflammatory molecule treatments. Indeed, DMD reflects the primary feature of myonecrosis associated with inflammation and OS [141]. During DMD, OS has a particular effect on protein damage and thiol oxidation, as demonstrated in mice [142]. The production of ROS occurs during myonecrosis after membrane disruption, or during activities of NAD(P)H oxidase, xanthine oxidase and the decoupling of NOSs [143]. DMD patients suffer from ischemia, impaired vasodilatation and a loss of NOS sarcolemma associated with an increase in ROS, which trigger calcium entry into mitochondria [144].

**Figure 3:**
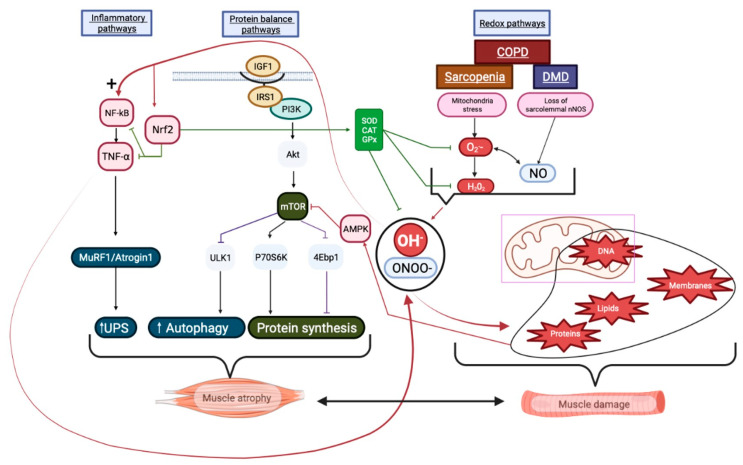
The roles of RONSs during skeletal muscle pathologies. Redox pathways in COPD, DMD and sarcopenia, mitochondria dysfunction and sarcomere NO leakage produce OS, which causes muscle damage leading to muscle atrophy. Protein balance pathways: the IGF1/PI3K/Akt/mTOR protein synthesis pathway is impacted by OS. Mitochondrial dysfunction due to the RONS active, AMPK, inhibiting mTOR and leading to rapid atrophy. Inflammatory pathways: inflammation is increased by OS via (+) NF-κB and TNF-α, activating UPS and resulting in muscle atrophy. In addition, OS triggers the release of antioxidant enzymes via Nrf2; however, SOD, CAT and GPx are insufficient to compensate for the overproduction of OH^−^ and ONOO^−^. According to the roles played by RONSs and their consequences, the described pathologies support OS-induced muscle atrophy and damage. Created using Biorender.com (accessed on 28 April 2023).

The pathologies described in this section are characterized by OS, leading to muscle atrophy and damage, the main mechanisms and signaling pathways which are reported in Figure 3.

## 5. Preventive Effects of Microalgal Biomasses and Their Bioactive Molecules in Muscle Oxidative Stress and Mitochondrial Functions

### 5.1. Microalgal Biomass

Two main animal models, rodents and fishes, have been studied to test the use of microalgal biomasses in muscle OS and mitochondrial functions.

The use of *Chlorella vulgaris* as a food supplement (150 and 300 mg·kg^−1^ for three months) in young and old rats has been shown to improve muscle mass, strength and function, which is explained by its potency to improve the OS management in skeletal muscles [145]. Another study investigating oxidative damage and metabolic changes induced by acute exercise in rats (6 h swimming) showed that a ten-day dietary supplementation with *Galdieria sulphuraria* (10 g·kg^−1^) reduced exercise-linked oxidative damage and mitochondrial dysfunction. Indeed, an increase in mitochondrial release rate of H_2_O_2_, and the liver and heart antioxidant enzyme activities have been observed. Moreover, a reduction in lipid oxidative damage was observed. These effects were proposed to be due to the high content of C-phycocyanin and glutathione in *Galdieria sulphuraria*, which are able to scavenge peroxyl radicals and contribute to phospholipid hydroperoxide metabolism, respectively [146]. In trained rats (high-intensity exercise for eight weeks), a dietary supplementation with *Spirulina platensis* (500 mg·kg^−1^) improved the antioxidant capacity, as well as reduced muscle damage and inflammation (C-reactive protein) [147]. Previously, these authors also showed that supplementation with *Spirulina platensis* (500 mg·kg^−1^·day^−1^) for eight weeks induced a significant improvement in exercise performance (time of execution), along with a significant decrease in muscle damage [147]. Using the seaweed of *Gracilaria asiatica* for supplementation (250 mg·kg^−1^ during 10 weeks) in OS induced by high-intensity resistance exercise, it has been reported that dietary fiber-rich algae could be a potential nutritional supplement to boost performance and prevent exercise-induced muscle damage through enhanced maximal carrying strength. The OS was reduced due to an increase in antioxidant status (SOD activity and GSH content), associated with a decrease in lipid peroxidation [148].

Fish (common carp) fed with 5% or 10% (*w*/*w*) *Chlorella vulgaris* biomass (rich in carotenoids as lutein) for 6 weeks, followed by daily treatment with chloramine-T (at concentration of 10 mg·L^–1^ for 1 hr in three consecutive days), showed increased GPx and CAT activities in muscles when compared to the control [149]. In another fish model, it was shown that feeding *Nile tilapia* with a microalgal mix containing *Nannochloropsis oculata*, *Schizochytrium* sp. and *Spirulina* sp. for 12 weeks was able to improve the oxidative status in muscles, characterized by a significant reduction in ROS, H_2_O_2_ and malondialdehyde (MDA) contents associated with the upregulation of GSH, GPx, CAT and SOD genes [150]. Supplementation with the microalgal strain, *Ascochloris* spp., in juvenile *Clarias gariepinus* fish, for an experimental period of 100 days, showed significantly higher glutathione S-transferase, CAT, SOD and lipid peroxidase activities in muscles [151]. Finally, catfish (*Rhamdia quelen*) supplemented for 60 days with 3% residual algal biomasses from *Acutodesmus obliquus*, a microalga rich in carotenoids and chlorophylls, increased muscle SOD activity, suggesting the potential antioxidant effect of pigments of *Acutodesmus obliquus* in improving the organism health status [152].

According to the reported data, microalgal biomasses have shown positive effects on the muscle redox status. This potential balance of the redox status produced by microalgae could have a major impact on muscle waste due to ageing, but also in COPD patients. Moreover, in DMD patients, the inflammation and NO leakage generated by muscle fiber necrosis or important fibrosis could be reduced by microalgal molecules. Supplementation with one of the microalgae could be interesting for recovery after a traumatic exercise session, and to observe the potential effects on muscle remodeling.

### 5.2. Carotenoids

The two main carotenoids present in microalgae are Fcx and Asx. Fcx has been reported to promote glucose uptake by skeletal muscle through the translocation of the glucose transporter, GLUT4, to membranes [153], but with different effects, depending on the muscle type [154,155]. In mice, the use of Fcx increased the weight of the tibialis anterior and gastrocnemius muscles, and decreases the OS in the tibialis anterior muscle through the phosphorylation of mTOR and suppression of the AMPK pathway [156,157].

When considering Asx, another xanthophyll well known for its beneficial effects on human health produced by the green alga, *Haematococcus pluvialis*, its potent antioxidant activity acts in the phospholipid bilayer membranes.

These beneficial effects of Asx and Fcx are related to their specific chemical strutures. Indeed, they are two oxycarotenoids, containing oxygen atoms and exhibiting a polyen chain with a conjugated carbonyl, an epoxide and hydroxyl groups on each end-cycle (Figure 1). This structure determines their potential biological functions and actions. Indeed, the central chain, including a system of conjugated double bonds, may carry cyclic end-groups, which can be substituted with oxygen-containing functional groups. This specific structure confers an effective reactive oxygen quencher role to Asx and Fcx, forming radical cations converted into stable compounds due to the electron transfer from vitamin E [15,158].

Used as food supplements during exercise experiments, the effect of Asx on ROS-targeted proteins has been shown in mouse skeletal muscles. Specifically, Asx was able to reduce the modification of carnitine palmitoyltransferase 1 induced by OS [159]. During exercise, Asx has been reported to increase mitochondria glutathione levels and limit OS in rat soleus muscles [160]. During long-term immobilization, feeding rats with Asx for 2 weeks induced an attenuation in atrophy of soleus muscle and suppressed myonuclear apoptosis [161]. Under these conditions, a decreased production of ROS and SOD-1, with an increased expression of vascular endothelial growth factor, were observed [162]. In a muscle-atrophied mouse model [163], Asx, used as a food supplement, has been reported to prevent muscle weight loss, with a decrease in myofiber size. In the soleus muscle, the authors observed a slight increase in H_2_O_2_. The upregulation of AMPK and peroxisome proliferators (PPAR-γ) favored mitochondrial biogenesis. In myotubes obtained from the soleus muscle, it has been shown that Asx was preferentially present in mitochondria and its effect suppressed ROS production.

Studies conducted in humans have revealed that four weeks of treatment with Asx were not enough to decrease the rate of lipid and carbohydrate oxidation during exercise [164]. In another study, Asx, provided through a *Haematococcus pluvialis* extract, was not reported to have any effect after 3 weeks of treatment on the markers of skeletal muscle injury, such as muscle soreness, creatine kinase activity and muscle performance [165].

It can be concluded that Asx and Fcx have shown their strong antioxidant effect on muscle, and specifically mitochondria. As in the cases of DMD, sarcopenic and COPD patients, mitochondria dysfunction is responsible for a lack of energy, reducing physical capacities, whereby microalgal carotenoids could improve energy production. Moreover, these molecules could limit muscle atrophy through a direct effect on the phosphorylation of mTOR or their antioxidant activity. Finally, as Asx increases the expression of AMPK and PPAR-γ, it can promote muscle adaptations and mitochondrial biogenesis during pathological or healthy conditions.

### 5.3. Omega-3 Polyunsaturated Fatty Acids

Although still elusive, the anabolic role of ω3-PUFAs on skeletal muscles is thought to be due to a reduction in proinflammatory cytokines and myosteatosis, an improvement in insulin sensitivity [166], MPS stimulation via the mTOR-p70S6K signaling pathway [167] and a decrease in mitochondrial ROS emission [168]. Muscle supplementation with ω3-PUFAs has been shown to increase their membrane phospholipid level related to the MPS and muscle loss prevention [169], that is linked to the preservation of adenosine diphosphate (ADP) sensitivity [170]. This is an important point, as ADP-stimulated oxidative phosphorylation reduces ROS production. Moreover, these preventive effects are also related to muscle mitochondrial functions, suggesting a close relation between the MPS and mitochondria bioenergetics [171] through the increased mRNA expression of transcription factors, such as PCG1-α, mitochondrial transcription factor A (TFAM) and Nrf1 [172], and the activation of AMPK [173]. As ω3-PUFAs are able to attenuate the generation of oxidative stress, their supplementation would improve muscle performance [174].

The effects of marine microalgae producing EPA and DHA, ω3-PUFAs well known to have benefits for human health, on mitochondrial functions and muscle pathologies will be described in the following section. Our review will focus on these molecules even through other ω3-PUFAs, such as docosapentaenoic acid (DPA, C22:5 ω3), which has been reported to have effects similar to EPA [175].

Clinical studies conducted in older men and women [176] have shown that dietary ω3-PUFA intake for 8 weeks (4 g/day) plays a role in muscle protein metabolism through an increased rate of MPS, as well as the protein kinase C and mTOR/p70S6K pathway activation. ω3-PUFA supplementation also increased muscle mass, strength and function in older adults, which may be in part transcriptionally regulated by the increased expression of the uncoupling protein 3 (UCP-3) and ubiquinol cytochrome-C reductase core protein 1 genes, involved in the regulation of mitochondrial functions. In opposition, pathways related to calpain-3 and ubiquitin-mediated proteolysis, and inhibition of the key anabolic regulator, mTOR, were reduced by ω3-PUFA supplementation [177]. Through reported observational studies and randomized controlled trials [168], muscle mass and physical activity have been associated with ω3-PUFA supplementation [178]. In sarcopenia, it has been recently proposed that ω3-PUFAs may be used for the prevention or treatment of this musculoskeletal disease [179]. However, the beneficial effects of ω3-PUFAs would depend on the dose used. Indeed, low daily doses of ω3-PUFAs (EPA and DHA, 0.225 g and 0.8 g, respectively) are not enough to have any effect on muscle mass, muscle strength and physical function in elderly people [180], in comparison with doses higher than 1 g of EPA and DHA [181]. The duration of dietary supplementation with ω3-PUFAs is also important, given that EPA and DHA supplementation for three to six months was enough to improve handgrip strength and muscle volume in the elderly population [181], while no change in results was observed after only three months of treatment [182]. In DMD, the intake of ω3-PUFAs as a dietary food supplement has been reported to increase MPS and decrease the inflammation cascade [183,184]. An explanation for an increased MPS by ω3-PUFAs is their role in the activation of the mTORC1/p70S6K1 signaling pathway and the downregulation of proteasome expression, leading to MPB suppression [185].

In rodent models, fish oil has been shown to reduce the decrease in the soleus muscle weight in the high fat (HF)-diet-fed group. During treatment, the expression of forkhead box O3 (FOXO3) and atrogin-1 proteins was also improved. The authors concluded that fish oil containing ω3-PUFAs was able to improve not only lipid imbalance, with activation of AMPK phosphorylation and expression of PPAR-γ and PGC-1α, but also muscle metabolism during HF diet [186].

In vitro studies conducted on C2C12 myotubes revealed that DHA was able to increase superoxide production, with a suppression of SOD activity, while EPA induced CAT activity [187]. In the same cell line, after stimulation with L-leucine, it has been shown that after treatment with EPA, MPS was increased in parallel to a decreased MPB, and an increased FOXO3a phosphorylation. In this study, the authors also reported that EPA and DHA were able to increase the phosphorylation of p70S6K, a key role in skeletal muscle atrophy [188]. After a treatment with palmitic acid (PAL, C16:0), a condition that induces lipotoxicity in the C2C12 cell line, EPA, DHA and DPA were found to prevent the effect of PAL, by promoting cell viability and differentiation of myoblasts into myotubes, that could be explained by an inhibition of PAL-induced proinflammatory cytokine expression [189]. In this study, the specificity of DPA was highlighted. Indeed, DPA maintained cell viability potentially via mitigating the loss of mitochondrial membrane integrity induced by PAL [190]. Using lipopolysaccharides (LPS) as a pro-inflammatory agent on C2C12 myoblasts, the co-treatment with EPA and DHA blunted the expression of IL-6 and TNF-α [189]. As proposed in clinical studies, these effects appear to be mediated by a restoration of the Akt/mTOR/FOXO3 pathway involved in the muscle differentiation process, as well as by an inhibition of the proinflammatory transcription factors activating protein-1 and NF-κB [191,192].

ω3-PUFAs, through DHA, EPA and DPA, have shown protective effects against energy metabolism diseases, allowing for protection against the development of diabetes and obesity in addition to sarcopenia, COPD and DMD. On the other hand, the action of ω3-PUFAs improves muscle mass, strength and inflammation upkeeps, without having an antioxidant effect against RONS. The ω3-PUFAs extracted from microalgae could have an anti-inflammatory role and be protective against metabolic alterations, which, when coupled with other microalgal molecules, could give synergetic effects.

### 5.4. Vitamins

Vitamin C, or ascorbic acid, is part of an exogenous, water-soluble, non-enzymatic group of antioxidants. Its antioxidant properties depend on its presence in different cell compartments [193]. Vitamin C is a molecule that can be used to prevent sarcopenia [194]. In humans, it has been reported that it was able to reduce age-related muscle loss [195] or increase muscle mass. Inversely, deficiency leads to muscle atrophy and a decrease in physical performance [196]. However, the effects of vitamin C during exercise on mitochondrial function are debatable. Indeed, in healthy men, vitamin C administration has been shown to decrease the benefits of endurance training on PGC-1α, despite maintaining whole-body adaptations and performance measures [197]. Moreover, it is unclear whether vitamin C can act directly on superoxide anions due to its bioavailability after supplementation and its subcellular location [67]. The use of vitamin C at high doses did not exert any effect on several skeletal–muscle parameters, such as PGC-1α and TFAM of PGC-related factor [83], or it may converted into a pro-oxidant molecule, specifically when combined with iron or copper, resulting in a hydroxyl radical level increase [193].

Vitamin E is a fat-soluble non-enzymatic antioxidant found in all cell membranes, the most common form being α-TOC [198]. As a lipophilic antioxidant, it protects membranes from damage, participating in cell immunity, and modulates signal transduction and gene expression in a redox-dependent and redox-independent manner, regulating cell functions relevant to its action and the prevention of diseases, such as cancer, atherosclerosis, inflammation or neurodegenerative diseases [199]. As vitamin C and carotenoids, vitamin E may attenuate sarcopenia, due to its ability to mitigate age-associated skeletal dysfunction and enhance muscle regeneration by modulating MPS [200]. At high doses, vitamin E may also inhibit signaling pathways triggered by the generated OS during exercise training [201]. However, as reported in a previous review taking into account dietary intakes and plasma nutrient levels, the effects of vitamin E are debatable relating the preservation of muscle mass and physical performance in specific populations where sarcopenia may be present [202]. Moreover, vitamin E does not seem to have any effect on muscle strength production after chronic strength training. Thus, its supplementation cannot potentiate muscle growth [104].

Vitamins E and A are recognized for their antioxidant properties in many diseases, including sarcopenia. Regarding COPD and DMD, they should have similar effects to counteract RONS. Thus, these vitamins being the compounds found in microalgae could also support the potential beneficial effects of microalgae on muscle diseases. On the other hand, one must be careful not to exceed these vitamins, as a contribution from microalgae alone may be sufficient to obtain only the positive effects.

### 5.5. Other Antioxidant Molecules

- Polysaccharides

Laminarin, a β-1,3 glucan is extracted from seaweed and is known for its effects on inflammation and oxidation [203]. It is also a dual regulator of apoptosis and cell proliferation, with antioxidant activity occurring through three mechanisms, including ROS scavenging, regulation of the antioxidant system and oxidative-stress-mediated signaling pathways [204]. In rat L6 myotubes, it has been reported that laminarin was able to activate the AMPK/p38MAPK (p38 mitogen-activated protein kinase) pathways in skeletal muscles, resulting in a better production of ATP and glucose uptake [205]. In fish muscles, β-glucans have been shown to decrease protein oxidation, lipid peroxidation and ROS levels. Specifically, β-glucans increased antioxidant enzyme activities, such as CAT, SOD or GPx, and decreased the expression of p66shc, a gene involved in the regulation of ROS levels [206]. In zebrafish, β-1,3 glucan from the alga *Poterioochromonas malhamensis* enhanced antioxidant capacity by reducing ROS contents [28]. In mice, polysaccharides (β-(1-3,4, and 6)-D-glucans) from the fungi *Ganoderma lucidum* [207] have been used to test their potential effects on the regulation of OS in skeletal muscles during swimming exercise. After 28 days of treatment, the polysaccharides increased the skeletal muscle activities of SOD, CAT and GPx, with a decrease in MDA levels, providing the downregulation of OS during exercise [208].

- Phenolic compounds

Very few studies have reported the effects of phenolic compounds on skeletal muscle physiology and mitochondrial functions. Among those produced by microalgae, gallic acid and ferulic acid can be mentioned.

Gallic acid is a benzoic acid that has been reported to prevent muscle decline due to ageing-associated oxidative stress. Low concentrations of gallic acid intake could delay skeletal muscle atrophy, as proposed in zebrafish embryos [209]. Studies conducted on C2C12 myotubes showed that gallic acid was associated with a significant increase in mitochondrial DNA and enzymatic activities, as well as an increase in mitochondrial turnover gene expression [210]. Gallic acid and gallate esters exert antioxidative properties with different mechanisms, such as ROS decrease and antioxidant enzyme activity increase [211]. Epigallocatechin gallate has been reported to upregulate PGC1-α expression in skeletal muscles, with an increased number of mitochondria. Moreover, it reduces the production of free radicals during exercise and inhibits the synthesis of slow-twitch muscle fibers, which prevent muscle damage [212].

Ferulic acid is a polyphenol widely known for its potential preventive effect on ageing or inflammation. During endurance exercise, administration of ferulic acid to mice for 12 days showed protection against the depletion of muscle enzymatic antioxidants, such as CAT, SOD and GPx [213]. In a cardiomyocyte OS cell model obtained after H_2_O_2_ treatment in mouse, ferulic acid protects the cardiomyocytes from OS [214]. In isolated rat psoas muscles, ferulic acid was able to increase GSH levels and SOD and CAT activities, in association with a decrease in NO levels [215]. To our knowledge, only one study has recently reported the effect of ferulic acid (trans, TFA) on skeletal muscle cells [216]. In this study, during hyperglycemia, TFA increased the activation of AMPK, and also increased the phosphorylation of acetyl-CoA carboxylase, suggesting that it could promote fatty acid oxidation. Moreover, under these experimental conditions, TFA reduced ROS and NO productions.

These other molecules extracted from microalgae show antioxidant effects via SOD, CAT, GPx and GSH, which could promote the redox profile in sarcopenia, DMD and COPD. Moreover, an increase of AMPK, PGC1-α and p38MAPK could improve mitochondrial biogenesis and glucose utilization, which, associated with a good antioxidant capacity, would limit defective mitochondria, allowing a compensated ATP supply.

**Figure 4:**
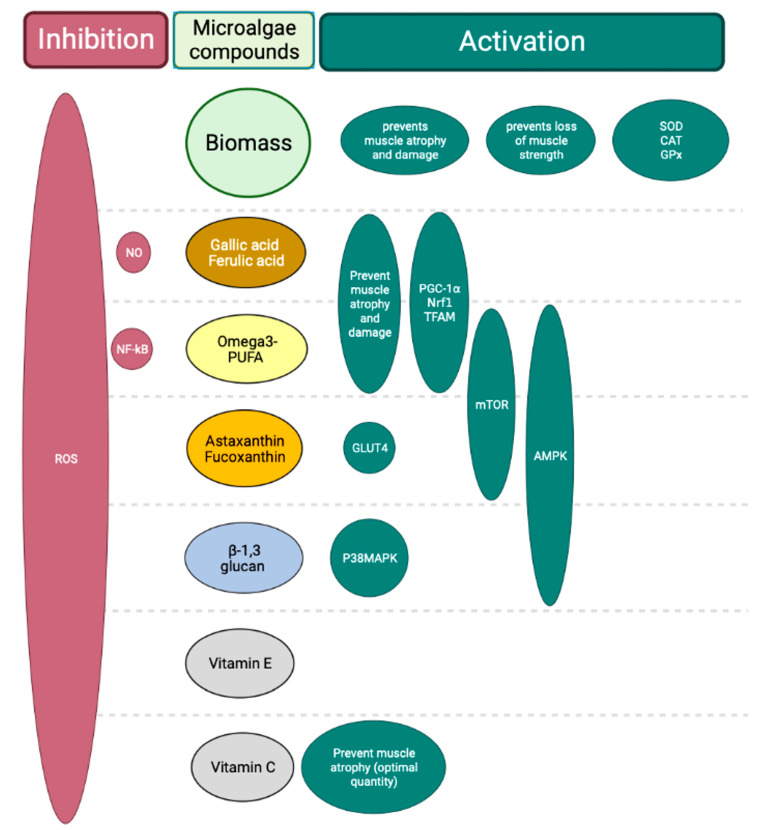
Microalgal effects described on skeletal muscle. Microalgal biomasses have been shown to prevent muscle atrophy and damage through contraction strength. Moreover, they increase antioxidant enzymatic activities, such as SOD, CAT and GPx. Microalgal compounds, such as gallic acid, ferulic acid and ω3-PUFAs, prevent muscle atrophy and damage, and activate mitochondrial biogenesis via the activation of Nrf1, TFAM and PGC-1α. The ω3-PUFA, Asx and Fcx are able to activate protein synthesis via the phosphorylation of mTOR. Then, these three molecules and β-1,3 glucan increase the activation of AMPK. All these reported microalgal molecules have an antioxidant activity against ROS. Created using Biorender.com (accessed on 28 April 2023).

In this review, the reported data have shown that microalgae and their bioactive molecules were able to have potential effects on the mitochondrial function and OS on skeletal muscle. The specific effects described in this review are synthesized in Figure 4.

## 6. Conclusions

Despite the limited scientific evidence of the influence of microalgae on skeletal muscles, to the authors’ knowledge, this paper is the first to provide a comprehensive review with considerations of the previous and most recent literature regarding the impact that supplementation with microalgal compounds could have on physical exercise and muscle pathologies under conditions of oxidative stress. Their antioxidant composition in the form of polysaccharides, vitamins, carotenoids and phenolic compounds associated with ω3-PUFA makes microalgae a cocktail that can bring synergistic effects on pathological or healthy skeletal muscle through an antioxidant capacity that needs to be further understood.

## Data Availability

Not applicable.
